# A nitrate-permeable ion channel in the tonoplast of the moss *Physcomitrella patens*

**DOI:** 10.1007/s00425-015-2250-3

**Published:** 2015-02-01

**Authors:** Mateusz Koselski, Halina Dziubinska, Aleksandra Seta-Koselska, Kazimierz Trebacz

**Affiliations:** 1 Department of Biophysics, Institute of Biology and Biochemistry, Maria Curie-Skłodowska University, Akademicka 19, 20-033 Lublin, Poland; 2 Department of Plant Physiology and Biotechnology, Institute of Biotechnology, The John Paul II Catholic University of Lublin, Konstantynow 1i, 20-708 Lublin, Poland

**Keywords:** Anion channels, Patch-clamp, *Physcomitrella*, Vacuole

## Abstract

**In this work, for the first time the activity of nitrate-permeable channels in the tonoplast of the moss** ***Physcomitrella patens*** **was recorded. The channels allowed nitrate flow in one direction—from the cytoplasm to the vacuole. Selectivity of nitrate over chloride of the channels was proved. The activity of the channels was dependent on cytoplasmic calcium, magnesium, and pH.**

A patch-clamp study carried out on the vacuolar membrane of the moss *Physcomitrella patens* has revealed that inhibition of cation-selective channels leads to disclosure of channels permeable to NO_3_^−^. These channels were inwardly rectifying and allowed anions to flow from the cytoplasm to the vacuole. After a decrease in the cytoplasmic NO_3_^−^ concentration, the current density recorded in the whole-vacuole configuration and amplitude of the currents flowing through single channels were reduced. Application of the NO_3_^−^ gradient caused a shift in the reversal potential (E_rev_) towards E_NO3−_, indicating NO_3_^−^ permeability. Research of the selectivity of the channels to Cl^−^ and NO_3_^−^ was also done; it indicated that Cl^−^ is less permeable than NO_3_^−^ (P_NO3_/P_Cl_ = 3.08). Measurements with different concentrations of cytoplasmic Ca^2+^ and Mg^2+^ revealed that the channel was activated by different concentrations of these ions—100 µM Ca^2+^ and 10 mM Mg^2+^. Calcium dependence of the channels was also modulated by a redox agent—DTT (dithiothreitol), which added on the cytoplasmic side, caused a reduction in the threshold of channel activation with cytoplasmic Ca^2+^. The NO_3_^−^ permeable channel was also pH dependent. A decrease in the cytoplasmic pH reduced the open probability of the channel; in turn, an increase in the vacuolar pH did not decrease ion channel activity but lowered its conductance.

**Electronic supplementary material:**

The online version of this article (doi:10.1007/s00425-015-2250-3) contains supplementary material, which is available to authorized users.

## Introduction

The tonoplast (vacuolar membrane) surrounding the largest, centrally located plant cell organelle (vacuole) is the object of electrophysiological investigations. Vacuoles are involved in maintenance of turgor in cells and accumulation of various molecules important for normal functioning of plant cells. Exchange of ions and substances between the vacuole and cytosol is achieved through ion channels, pumps, and transporters. The simplicity of vacuole isolation and the size of these objects facilitate application of different electrophysiological techniques, including the patch-clamp technique. This technique allows analysis of ion fluxes through ion channels located in the tonoplast. This method was employed in investigations of many types of ion channels in tonoplasts of various plants, primarily cation channels, and their function in the cell. There are considerably fewer papers concerning anion channels in the tonoplast, although the channels play an equally important role in osmoregulation, signalling, and maintenance of cell homeostasis. An important group of proteins involved in anion transport is represented by a family of chloride channels (CLCs) located in cell membranes in both prokaryotes and eukaryotes. Among the wide family of these transporters, some are chloride channels, like ClC-0 found in the torpedo-fish (Miller and White 1980) and mammalian ClC-1 (Steinmeyer et al. 1991), whereas others like ClC-ecl from *Escherichia coli* (Accardi and Miller 2004) and mammalian ClC-4 and ClC-5 (Picollo and Pusch 2005) are known as Cl^−^/H^+^ exchangers.

CLC genes were the first identified genes encoding anion channels in *Nicotiana tabacum* (Lurin et al. 1996) and *Arabidopsis* (Hechenberger et al. 1996). Four novel members of the CLC family cloned in *Arabidopsis* are homologous to the chloride channel gene (*CLC*-*Nt1*) from tobacco (Lurin et al. 1996). Similarly, *OsCLC1* and *OsCLC2* genes, which encode the proteins of chloride channels in vacuolar membranes in rice, were found to be homologous to the tobacco *CLC*-*Nt1* gene (Nakamura et al. 2006). Another gene (*GmCLC1*) encodes a chloride channel localised in the soybean tonoplast (Li et al. 2006).

Currently, seven genes encoding CLC proteins serving the function of anion channels or transporters have been identified in the genome of *Arabidopsis* (Hechenberger et al. 1996; Marmagne et al. 2007; Lv et al. 2009; von der Fecht-Bartenbach et al. 2010). The AtCLCa protein located in the vacuole acts as a proton-nitrate exchanger. It contributes up to 50-fold increase in nitrate accumulation in the vacuole relative to the cytoplasm (De Angeli et al. 2006). Achievement of such a high nitrate gradient would not be possible at passive transport through the channels. Patch-clamp investigations have shown that AtCLCa acts in the tonoplast as an exchanger mediating an influx of two nitrate anions into the vacuole and an efflux of one proton from the vacuole into the cytoplasm. The vacuolar H^+^/NO_3_^−^ exchanger properties are also exhibited by AtCLCb; however, in the case of this protein, the exact coupling ratio has not been yet determined (von der Fecht-Bartenbach et al. 2010). The vacuolar location of two other proteins, AtCLCc and AtCLCg, has been experimentally evidenced (Lv et al. 2009; Jossier et al. 2010), but there is still lack of data allowing classification of these proteins to a particular type of transporters—anion channels and H^+^/Cl^−^ or H^+^/NO_3_^−^ exchangers. The well-known X-ray structure of the ClC-ec1 homologue can be helpful for qualification of these two proteins to channels or to exchangers (Accardi and Miller 2004; Accardi et al. 2005). According to this research, one of the two glutamic acid residues (E203) present in all known CLC exchangers (including AtCLCa-d, g) is required for proton exchange and is proposed to be a mark for distinguishing channels from exchangers.

Considering the ways of nitrate uptake by plant cells, one member of NRT2 (NitRate Transporter) family should be mentioned—NRT2.7. In *Arabidopsis thaliana,* this transporter (AtNRT2.7) is located in the tonoplast of seeds and takes part in nitrate loading into the vacuole (Chopin et al. 2007).

Knowledge of the basis of anion transport and anion selectivity in plant tonoplasts at different systematic levels will allow determination of the evolution of anion transport systems. *Physcomitrella patens*, a plant with a fully identified genome and with a systematic position between bacteria and higher plants, would be a useful link in tracing possible changes in anion transport through the tonoplast.

## Materials and methods

### Plant material

A moss *Physcomitrella patens*, obtained by courtesy of Prof. Dr. Ralf Reski (University of Freiburg, Germany), was grown in a growth chamber at 22 °C with light intensity of 60 µmol m^−2^ s^−1^ under a 16/8 h light/dark photoperiod. The cultivation of the moss was carried out in Petri dishes filled with solid KNOP medium (Reski and Abel 1985). The moss culture was maintained by passaging of gametophytes into a fresh medium.

### Vacuole isolation

Vacuoles were isolated using a non-enzymatic method described by Trebacz and Schönknecht (2000). Before the experiments, leaves of the gametophytes were placed in a plasmolysing medium containing 650 mM sorbitol and different ions, depending on the experiment. After 20–30 min, several leaves were cut and placed in the measuring chamber containing a solution with lower than earlier osmotic pressure (about 350 mOsm kg^−1^). Deplasmolysis of the cells caused release of a few protoplasts from the cutting-destroyed cell walls. After a few minutes, some of the protoplasts ruptured which allowed release of the vacuoles.

### Patch-clamp experiments

The experiments were carried out in two patch-clamp configurations—whole-vacuole and cytoplasm-out. The micropipettes made from borosilicate tubes (Kwik-Fil TW150-4; WPI, Sarasota, FL, USA) were pulled and polished by a DMZ-Universal Puller (Zeitz-Instruments, Martinsried, Germany). An Ag/AgCl reference electrode was filled with 100 mM KCl and contacted with the bath solution by a ceramic porous bridge. Osmolarity of the solutions was checked using a cryoscopic osmometer (Osmomat 030; Gonotec, Berlin, Germany). The experiments were recorded using an EPC-10 amplifier (Heka Electronik, Lambrecht, Germany), running with the Patchmaster software (Heka Electronik). The sample frequency was 10 kHz with a 2 kHz filter. The sign of the tonoplast voltage was the same as that proposed by Bertl et al. (1992). The bath solutions were exchanged before recording using a peristaltic pump (ISM796B; Ismatec, Wertheim, Germany). The values of voltages applied in the experiments include the magnitude of the liquid junction potentials measured according to the method described by Amtmann and Sanders (1997).

### Analysis of the results

Current/voltage (I/V) and current density/voltage (J/V) characteristics were made in SigmaPlot 9.0 (Systat Software Inc.). The Gaussian fits of the histograms were made in GRAMS/AI 8.0 (Spectroscopy Software). The area under the Gaussian peaks was used in calculations of the channel open probability. Ion activities were taken into account in calculations of the reversal potentials for the ions (E_rev_). Activities of the ions were also used in calculations of the permeability ratio obtained from the Goldman-Hodgkin-Katz equation. Due to lack of linear characteristics of the I/V curves, the unitary conductance of the channels was calculated as a current/voltage ratio determined at the most extreme voltage applied. The number of the experimental repeats (*n*) indicates the number of tested vacuoles or tonoplast patches.

### Solution composition

Solutions used for recording the SV channels contained 200 mM NaNO_3_, 2 mM CaCl_2_, 2 mM MgCl_2_, pH 7 (buffered by HEPES/TRIS) on the cytoplasmic side and 200 mM NaNO_3_, 2 mM CaCl_2_, 2 mM MgCl_2_, pH 5 (buffered by MES/TRIS) on the vacuolar side. Reduction of currents carried by SV channels was achieved by replacement of the solution on the cytoplasmic side with 200 mM HNO_3_, 2 mM CaCl_2_, 2 mM MgCl_2_ (buffered by 160 mM BTP). NO_3_^−^ permeable channels were recorded in solutions containing 200 mM HNO_3_, 2 mM CaCl_2_, 2 mM MgCl_2_, pH 5 (buffered by 101 mM BTP) on the vacuolar side, and 200 mM HNO_3_, 2 mM CaCl_2_, 2 mM MgCl_2_, pH 7 (buffered by 160 mM BTP). The selectivity of the channels was studied by replacement of the solution on the cytoplasmic side with 20 mM HNO_3_, 2 mM CaCl_2_, 2 mM MgCl_2_, pH 7 (buffered by 16 mM BTP). Solutions on the cytoplasmic side were also replaced in measurements of the dependence of the channel activity on cytoplasmic Ca^2+^ and Mg^2+^ and pH. Ca^2+^ and Mg^2+^ dependence was studied in 200 mM HNO_3_, 2 mM EGTA, 2 mM MgCl_2_, pH 7 (buffered by 148 mM BTP) mixed with a suitable amount of CaCl_2_ or MgCl_2_, the concentrations of which were calculated in Ca-EGTA Calculator v1.3 (http://maxchelator.stanford.edu/CaEGTA-TS.htm) and Ca–Mg-ATP-EGTA Calculator v1.0 (http://maxchelator.stanford.edu/CaMgATPEGTA-NIST.htm), respectively. Measurements of pH dependence were carried out in 200 mM HNO_3_, 2 mM CaCl_2_, 2 mM MgCl_2_, buffered by 131 mM BTP (pH 6.5) or by 113 mM BTP (pH 6.0). The experiments aimed at determination of Cl^−^ over NO_3_^−^ selectivity were begun in 200 mM HCl, 2 mM CaCl_2_, 2 mM MgCl_2_, pH 5 (buffered by 82 mM BTP) on the vacuolar side and 200 mM HCl, 2 mM CaCl_2_, 2 mM MgCl_2_, pH 7 (buffered by 128 mM BTP) on the cytoplasmic side. Next, the solution on the cytoplasmic side was replaced with 200 mM HNO_3_, 2 mM CaCl_2_, 2 mM MgCl_2_, pH 7 (buffered by160 mM BTP).

## Results

In our previous study (Koselski et al. 2013), we demonstrated that two different cation-selective channels were active in the vacuole of the moss *Physcomitrella patens*—SV channels permeable to Na^+^, K^+^, Ca^2+^, Mg^2+^, and K^+^- selective VK channels. In the present study, we reduced the activity of SV and VK channels by elimination of cations which can flow through these channels. Since there are reports on plant vacuolar anion channels indicating higher permeability to NO_3_^−^ than to Cl^−^ (De Angeli et al. 2006; von der Fecht-Bartenbach et al. 2010), we used NO_3_^−^ as the main anion.

**Fig. 1:**
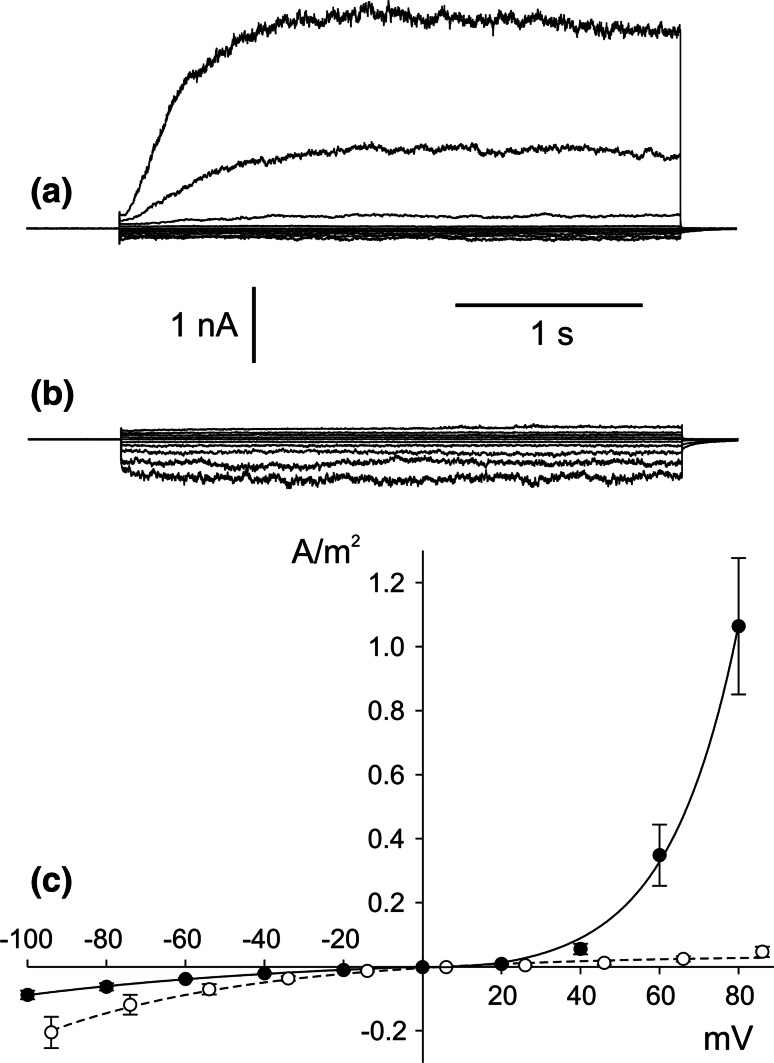
Activation of negative currents concomitant with reduction of SV channels activity. **a** Example of whole-vacuole recordings obtained in 200 mM NaNO_3_, 2 mM CaCl_2_, 2 mM MgCl_2_, pH 7 (buffered by HEPES/TRIS) in the bath and 200 mM NaNO_3_, 2 mM CaCl_2_, 2 mM MgCl_2_, pH 5 (buffered by MES/TRIS) in the pipette. **b** Recordings obtained on the same vacuole as in **a** after replacement of the bath solution with 200 mM HNO_3_, 2 mM CaCl_2_, 2 mM MgCl_2_, pH 7 buffered by 160 mM BTP. **c** J/V curves obtained in the same conditions as in **a** (*closed circles* and *solid line*, *n* = 4) and **b** (*open circles* and *dashed line*, *n* = 4), respectively. Recordings were obtained by application of 0.5 s holding voltage (0 mV for **a** and 6 mV for **b**), then 3 s test voltages with 20 mV steps (from −100 to 80 mV for **a** and from −94 to 86 mV for **b**), and 0.3 s pulse (0 mV for **a** and 6 mV for **b**) after test voltage

**Fig. 2:**
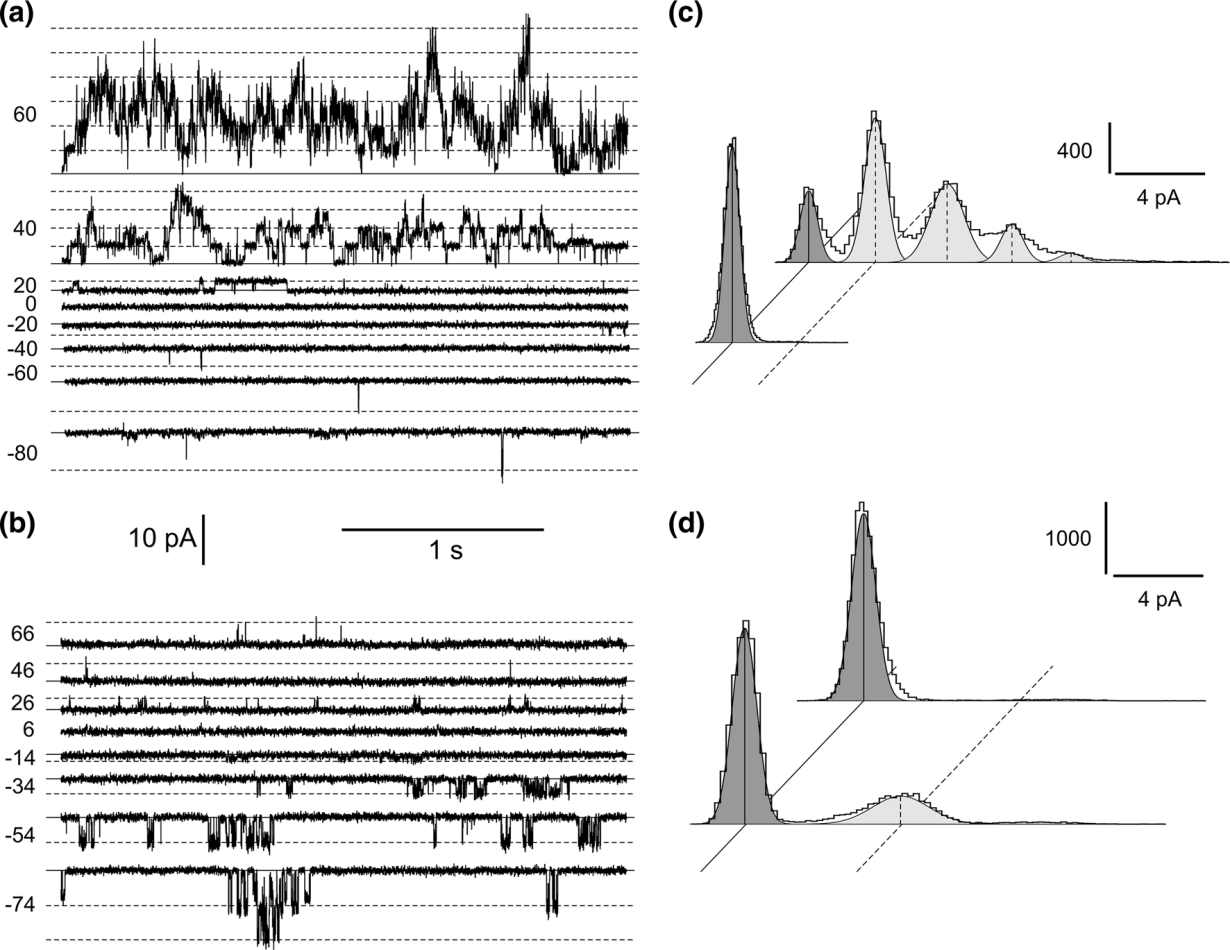
Activation of ion currents recorded at negative voltages concomitant with reduction of SV currents. **a** Example of cytoplasm-out recordings obtained in 200 mM NaNO_3_, 2 mM CaCl_2_, 2 mM MgCl_2_, pH 7 (buffered by HEPES/TRIS) in the bath and 200 mM NaNO_3_, 2 mM CaCl_2_, 2 mM MgCl_2_, pH 5 (buffered by MES/TRIS) in the pipette. The *solid line* indicates the closed state of the channels and the *dashed line* the open states. Values of holding voltages were placed on the left side of the traces. **b** Recordings obtained on the same vacuole as in **a** after replacement of bath solution with 200 mM HNO_3_, 2 mM CaCl_2_, 2 mM MgCl_2_, pH 7 buffered by 160 mM BTP. **c** Amplitude histograms indicating the number of sample points (*vertical bar*) and a certain current amplitude (*horizontal bar*), based on recordings from four patches obtained at +40 mV in conditions as in **a** (upper histogram) and at +46 mV in conditions as in **b** (lower histogram), respectively. The *diagonal solid line* indicates the closed state, and the *dashed line* the open state. **d** Amplitude histograms based on recordings from four patches obtained at −80 mV in conditions as in **a** (upper histogram) and **b** (lower histogram), respectively

The first step in our research was studying the effect of SV channel activity reduction by replacement of cytoplasmic 200 mM NaNO_3_ with 200 mM HNO_3_ (Fig. 1). Together with elimination of cytoplasmic Na^+^, we also decided to reduce SV channel activity using BTP—an impermeable cation—as a buffer. In the whole-vacuole configuration, elimination of cytoplasmic Na^+^ caused reduction of currents carried by SV channels (from 1.06 ± 0.21A/m^2^ recorded at +80 mV, *n* = 4 to 0.05 ± 0.02 A/m^2^ recorded at +86 mV, *n* = 4) but also an increase in the current density recorded at negative voltages (from 0.09 ± 0.01A/m^2^ recorded at −100 mV, *n* = 4 to 0.2 ± 0.05 A/m^2^ recorded at −94 mV, *n* = 4). The above-mentioned phenomenon was caused by changes in the number of active ion channels, which was confirmed by the single channel recordings carried out in the cytoplasm-out configuration (Fig. 2). The amplitude histograms based on the recordings obtained at +40 mV, which correspond to the activity of SV channels (Fig. 2c) showed complete reduction of the channel activity after replacement of NaNO_3_ with HNO_3_. In turn, at negative voltages, the lack of Na^+^ on the cytoplasmic side revealed activation of ion currents (Fig. 2b, d). The amplitude histograms based on the recordings of the channels at −74 mV in the absence of cytoplasmic Na^+^ (Fig. 2d, lower histogram) and SV channels recorded at +40 mV in the presence of cytoplasmic Na^+^ (Fig. 2c, upper histogram) allow determination of the number of channels active in one patch and also an open probability and unitary conductance. The average number of SV channels active in one patch (at +40 mV) was higher than that of the other channels active at −74 mV (4 channels in respect to 1). The number of active channels did not reflect the open probability, because this parameter was similar for both channels (0.21 for SV channels recorded at +40 mV and 0.25 for channels recorded at −74 mV). A comparison of the differences between the positions of the Gaussian peak centres indicated different unitary conductance of the channels, which at −74 mV reached 86.75 pS and at +40 mV −72.75 ± 0.11 pS (*n* = 4).

**Fig. 3:**
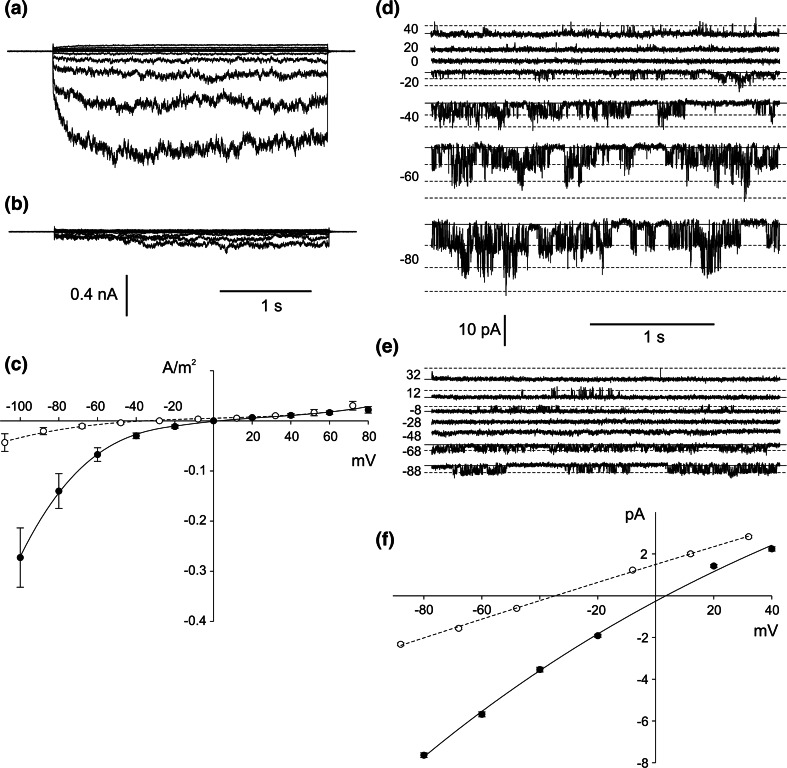
Activity of negative currents at different cytoplasmic HNO_3_ concentrations. **a** Whole-vacuole currents obtained in 200 mM HNO_3_, 2 mM CaCl_2_, 2 mM MgCl_2_, pH 7 (buffered by 160 mM BTP) in the bath and 200 mM HNO_3_, 2 mM CaCl_2_, 2 mM MgCl_2_, pH 5 (buffered by 101 mM BTP) in the pipette. **b** Whole-vacuole currents obtained after replacement of the bath solution with 20 mM HNO_3_, 2 mM CaCl_2_, 2 mM MgCl_2_, pH 7 (buffered by 16 mM BTP). **c** J/V curves obtained in the same conditions as in **a** (*closed circles* and *solid line*, *n* = 5) and **b** (*open circles* and *dashed line*, *n* = 5), respectively. **d**, **e** Cytoplasm-out recordings obtained under the same conditions as in **a** and **b**, respectively. **f** I/V curves obtained under the same conditions as in **d** (*closed circles* and *solid line*, *n* = 6) and **e** (*open circles* and *dashed line*, *n* = 6), respectively. Whole-vacuole recordings were obtained by application of 0.5 s holding voltage (0 mV in **a** and −8 mV in **b**), then 3 s test voltages with 20 mV steps (from −100 to 80 mV in **a** and from −108 to 72 mV in **b**), and 0.3 s pulse (0 mV in **a** and −8 mV in **b**) after test voltage

The whole-vacuole recordings obtained in symmetrical (in the patch pipette and in the medium) concentrations of NO_3_^−^ allowed observation of inward rectification and slow activation of the channels (Fig. 3a). The amplitude of the whole-vacuole currents was dependent on the cytoplasmic concentration of NO_3_^−^, which after tenfold reduction, caused a decrease in the negative currents from 0.27 ± 0.06 A/m^2^ recorded at −100 mV (*n* = 5) to 0.04 ± 0.02 A/m^2^ recorded at −108 mV (*n* = 5) (Fig. 3b, c). Besides the changes in the whole-vacuole current amplitudes, there was also a shift in the reversal potential towards the equilibrium potential for NO_3_^−^ (E_NO3_) (Fig. 3c), although determination of the reversal potential in this configuration was inaccurate due to strong rectification. The decrease in the whole-vacuole currents was caused by a decrease in the current flowing through a single channel, confirmed by the cytoplasm-out recordings (Fig. 3d, e, f). The value of the channel conductance decreased from 95.5 ± 1.37 pS recorded at −80 mV (*n* = 6) to 29.12 ± 0.87 pS recorded at −88 mM (*n* = 6). The cytoplasm-out recordings carried out in the NO_3_^−^ gradient confirmed NO_3_^−^ permeability of the channels because the reversal potential obtained from the I/V curve was shifted towards E_NO3_ (whose value in the gradient of NO_3_^−^ amounts to −51.6 mV) from 3.5 to −34.9 mV (Fig. 3f). The cytoplasm-out recordings also showed dependence of single channel open probability on the cytoplasmic NO_3_^−^ concentration. At 200 mM cytoplasmic NO_3_^−^, the open probability of single channels reached 0.39 at −80 mV and dropped to 0.19 at −88 mV after tenfold reduction of the NO_3_^−^ concentration (Supplementary Material). The decrease in the open probability recorded at the low cytoplasmic NO_3_^−^ concentration can be explained by a shift of the activation voltage toward negative voltages.

**Fig. 4:**
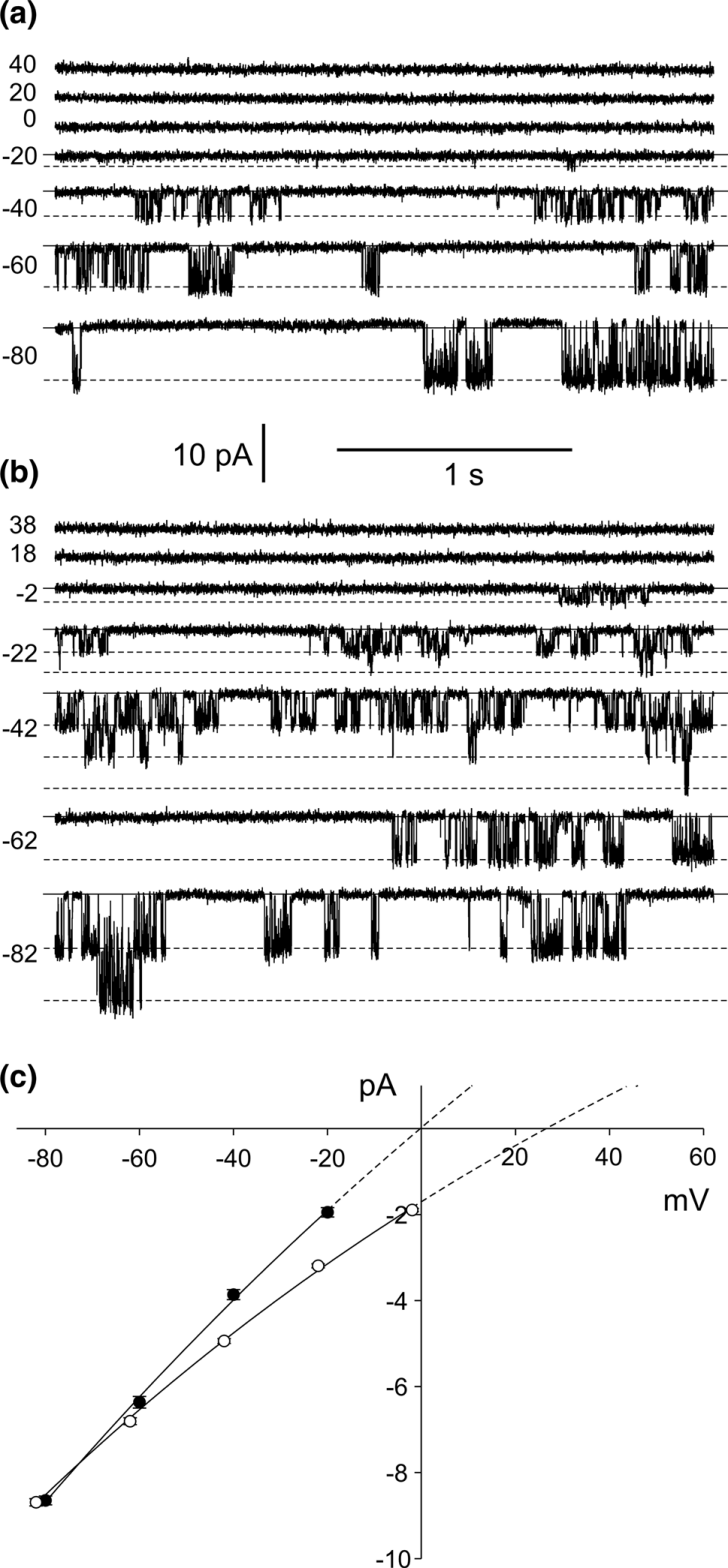
NO_3_^−^ over Cl^−^ selectivity of the channels. **a** Cytoplasm-out recordings obtained in 200 mM HCl, 2 mM CaCl_2_, 2 mM MgCl_2_, pH 7 (buffered by 128 mM BTP) in the bath and 200 mM HCl, 2 mM CaCl_2_, 2 mM MgCl_2_, pH 5 (buffered by 82 mM BTP) in the pipette. **b** Cytoplasm-out recordings obtained after replacement of the bath solution with 200 mM HNO_3_, 2 mM CaCl_2_, 2 mM MgCl_2_, pH 7 (buffered by 160 mM BTP). **c** I/V curves obtained under the same conditions as in **a** (*closed circles*, *n* = 5) and **b** (*open circles*, *n* = 5), respectively

Besides NO_3_^−^, also Cl^−^ permeability of the channels was studied. In the symmetrical concentration of these anions (Fig. 4a), as in the case of symmetrical NO_3_^−^, channel activity was recorded only at negative voltages. The unitary conductance of the channels at −80 mV equalled 108.1 ± 1.3 pS (*n* = 5). This value was close to the unitary conductance obtained in the symmetrical concentration of NO_3_^−^, which reached 95.5 ± 1.4 pS at −80 mV (*n* = 6). To study Cl^−^/NO_3_^−^ selectivity during cytoplasm-out recordings, Cl^−^ was replaced with NO_3_^−^ (Fig. 4b). In such conditions, a shift in the reversal potential to positive values was observed (from −0.2 to 26.6 mV, Fig. 4c), indicating NO_3_^−^ over Cl^−^ selectivity. The NO_3_^−^/Cl^−^ permeability ratio of the channels determined by the Goldman-Hodgkin-Katz equation amounted to 3.08.

**Fig. 5:**
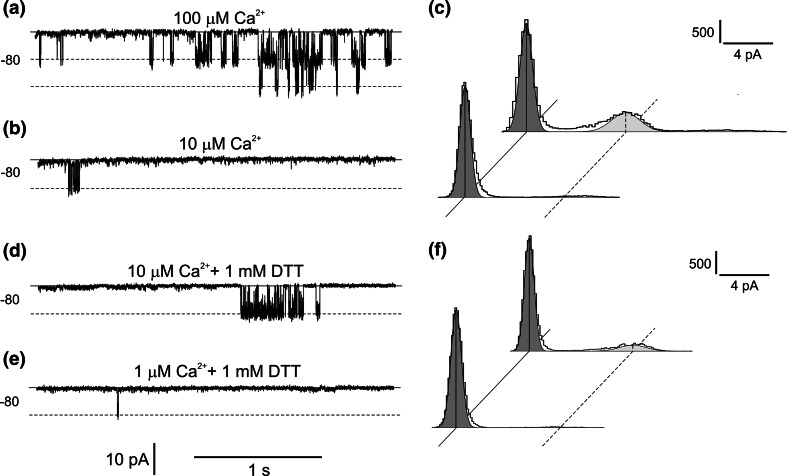
Dependence of the channel activity on the cytoplasmic Ca^2+^ concentration recorded in the absence (**a**, **b**) and presence (**d**, **e**) of cytoplasmic DTT. **a** Cytoplasm-out recordings obtained in the presence of 100 µM free Ca^2+^ at −80 mV. The bath solution contained 200 mM HNO_3_, 2 mM EGTA, 2.08 mM CaCl_2_, 2 mM MgCl_2_, pH 7 (buffered by 148 BTP), and the pipette 200 mM HNO_3_, 2 mM CaCl_2_, 2 mM MgCl_2_, pH 5 (buffered by 101 BTP). **b** Cytoplasm-out recordings obtained after reduction of the free cytoplasmic Ca^2+^ concentration to 10 µM by replacement of the bath solution with 200 mM HNO_3_, 2 mM EGTA, 1.84 mM CaCl_2_, 2 mM MgCl_2_, pH 7 (buffered by 148 BTP). **c** Amplitude histograms based on recordings from four patches obtained at −80 mV in conditions as in **a** (upper histogram), and **b** (lower histogram), respectively. **d** Cytoplasm-out recordings obtained in the presence of cytoplasmic 1 mM DTT and 10 µM free Ca^2+^. The bath solution contained 200 mM HNO_3_, 1 mM DTT, 2 mM EGTA, 1.84 mM CaCl_2_, 2 mM MgCl_2_, pH 7 (buffered by 148 BTP), and the pipette the same solution as used in **a**. **e** Cytoplasm-out recordings obtained after reduction of the free cytoplasmic Ca^2+^ concentration to 1 µM by replacement of the bath solution with 200 mM HNO_3_, 1 mM DTT, 2 mM EGTA, 1.04 mM CaCl_2_, 2 mM MgCl_2_, pH 7 (buffered by 148 BTP). **f** Amplitude histograms based on recordings from four patches obtained at −80 mV under conditions as in **d** (upper histogram), and **e** (lower histogram), respectively

**Fig. 6:**
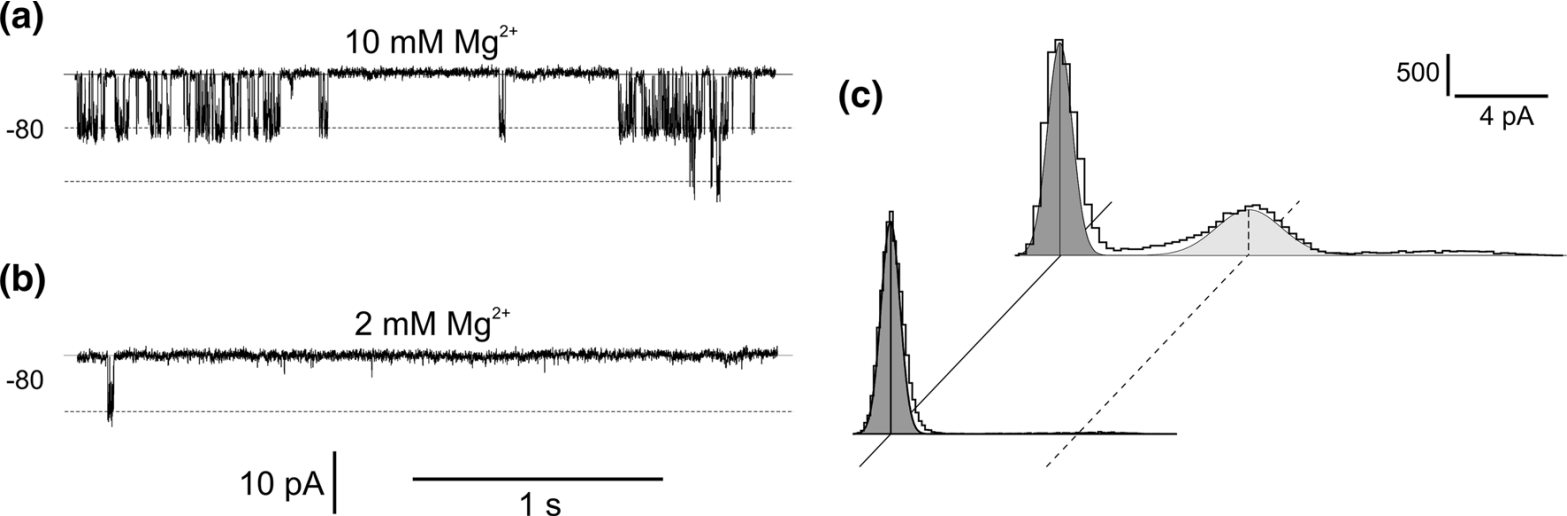
Dependence of the channel activity on the cytoplasmic Mg^2+^ concentration. **a** Cytoplasm-out recordings obtained in the presence of 10 mM free Mg^2+^ at −80 mV. The bath solution contained 200 mM HNO_3_, 2 mM EGTA, 10.44 mM MgCl_2_, 2 mM, pH 7 (buffered by 148 BTP), and the pipette 200 mM HNO_3_, 2 mM CaCl_2_, 2 mM MgCl_2_, pH 5 (buffered by 101 BTP). **b** Cytoplasm-out recordings obtained after reduction of the cytoplasmic Mg^2+^ concentration to 2 mM by replacement of the bath solution with 200 mM HNO_3_, 2 mM EGTA, 2.11 mM MgCl_2_, pH 7 (buffered by 148 BTP). **c** Amplitude histograms based on recordings from four patches obtained at −80 mV in conditions as in **a** (upper histogram), and **c** (lower histogram), respectively

The activity of the NO_3_^−^ permeable channels was dependent on cytoplasmic Ca^2+^ and Mg^2+^. The dependence of the channels on cytoplasmic Ca^2+^ was studied at 10 and 100 µM concentrations of this ion (Fig. 5a, b, c). Changes in the cytoplasmic Ca^2+^ concentration from 100 to 10 µM caused reduction of the channel activity by decreasing the open probability from 0.28 to 0.02 (Fig. 5c). The calcium sensitivity of the channel was modulated by cytoplasmic DTT (Fig. 5d, e, f) —a reducing agent that is effective in activation of voltage- and calcium-dependent channels including plant SV channels (Carpaneto et al. 1999; Paganetto et al. 2001; Scholz-Starke et al. 2004). Application of 1 mM DTT on the cytoplasmic side caused an increase in the open probability recorded in the presence of 10 µM cytoplasmic Ca^2+^ (Fig. 5d) from 0.02 (Fig. 5c, lower histogram) to 0.13 (Fig. 5f, upper histogram). The presence of cytoplasmic DTT was not sufficient to maintain the channel activity after reduction of the cytoplasmic Ca^2+^ concentration to 1 µM (Fig. 5e), resulting in a decrease in the open probability to 0.01 (Fig. 5f, lower histogram). The results show that under the experimental conditions used here, cytoplasmic Ca^2+^ activates the channels in a non-physiological concentration, because the Ca^2+^ concentration in the cytoplasm of plant cells is typically maintained at approx. 200 nM (Bush 1995). In contrast to Ca^2+^, cytoplasmic Mg^2+^ acts on the channel activity in a physiological range of concentrations (Fig. 6), since this ion occurs in the cytoplasm of leaf cells from higher plants at concentrations from 2 to 10 mM (Leigh and Wyn Jones 1986). The measurements with 2 and 10 mM Mg^2+^ proved that the 10 mM concentration of this ion was efficient to activate the channels. In such concentrations, the open probability of the channels amounted to 0.36 and was reduced to 0.012 in the presence of 2 mM Mg^2+^ (Fig. 6c).

**Fig. 7:**
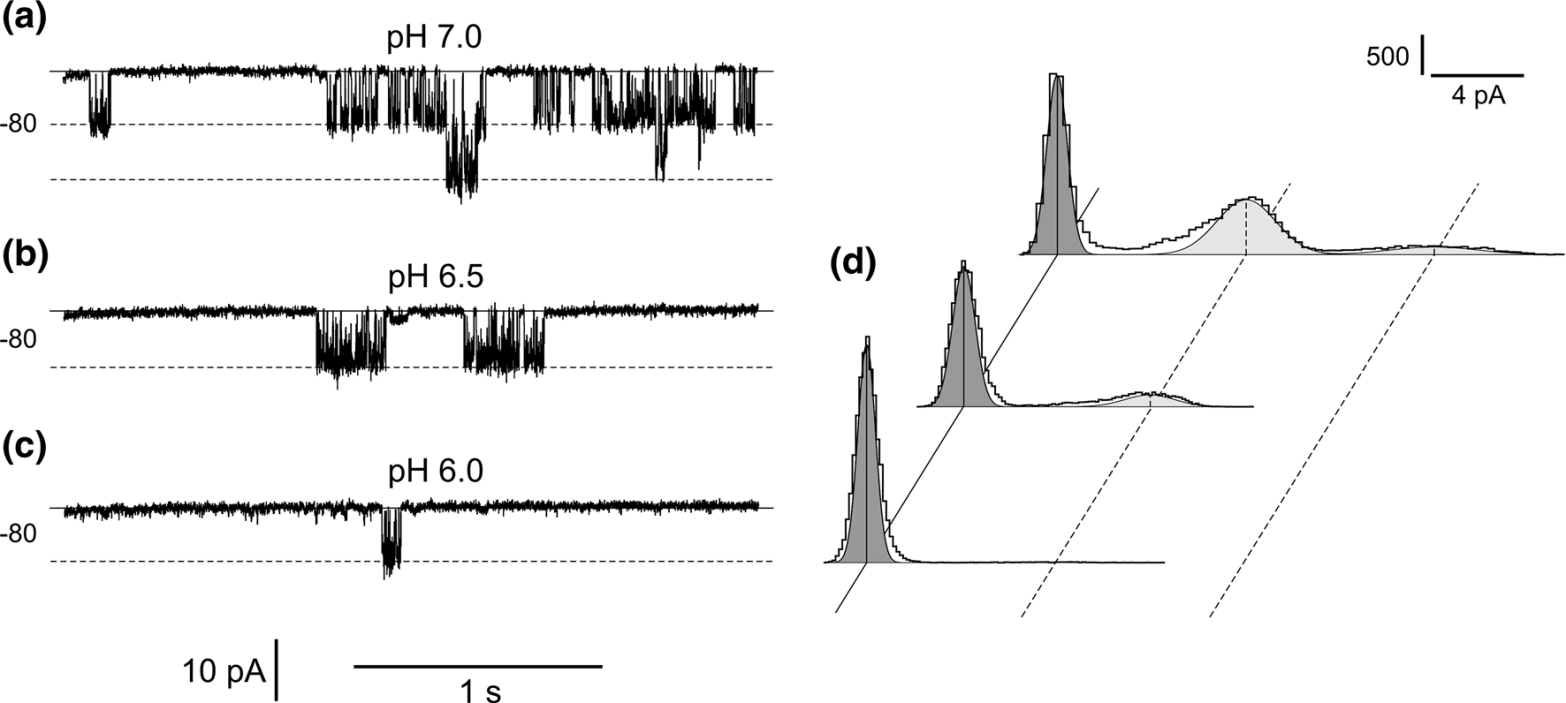
Dependence of the channel activity on cytoplasmic pH. **a** Cytoplasm-out recordings obtained at −80 mV in 200 mM HNO_3_, 2 mM CaCl_2_, 2 mM MgCl_2_, pH 7 (buffered by 160 mM BTP) in the bath and 200 mM HNO_3_, 2 mM CaCl_2_, 2 mM MgCl_2_, pH 5 (buffered by MES/TRIS) in the pipette. **b**, **c** Cytoplasm-out recordings obtained after reduction of the cytoplasmic pH to 6.5 (by replacement of the bath solution with 200 mM HNO_3_, 2 mM CaCl_2_, 2 mM MgCl_2_, 131 mM BTP) and 6.0 (by replacement of the bath solution with 200 mM HNO_3_, 2 mM CaCl_2_, 2 mM MgCl_2_, 113 mM BTP), respectively. **d** Amplitude histograms based on recordings from four patches obtained at −80 mV in conditions as in **a** (upper histogram), **b** (middle histogram) and **c** (lower histogram), respectively

**Fig. 8:**
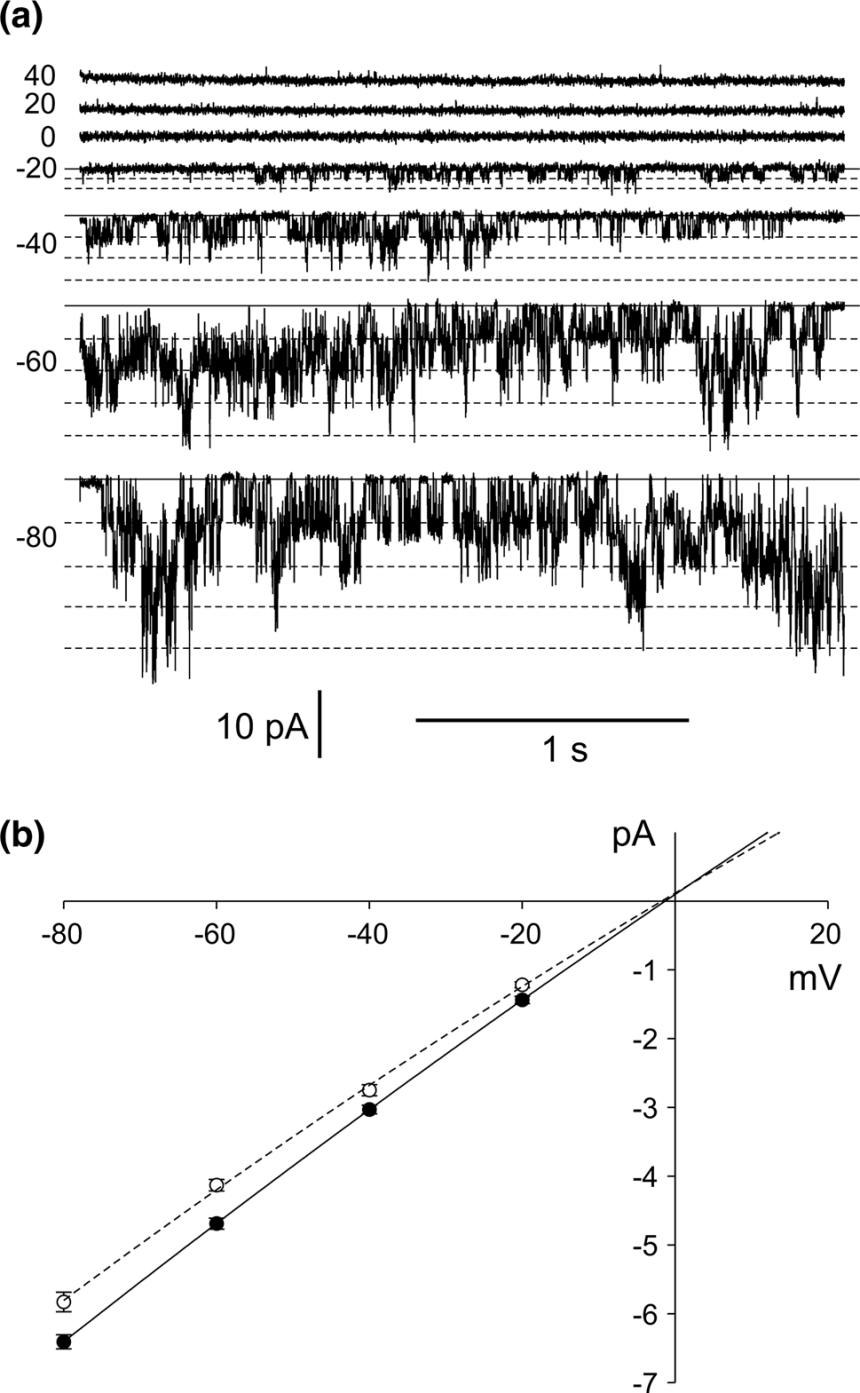
Single channel activity recorded at high vacuolar pH. **a** Cytoplasm-out recordings obtained at symmetrical (in the bath and in the pipette) 200 mM HNO_3_, 2 mM CaCl_2_, 2 mM MgCl_2_, pH 7 (buffered by 160 mM BTP). **b** I/V curves obtained under the same conditions as in **a** (*closed circles* and *solid line*, *n* = 9) and after increasing the vacuolar pH to 8 by application of 200 mM HNO_3_, 2 mM CaCl_2_, 2 mM MgCl_2_, 193 mM BTP in the pipette (*open circles* and *dashed line*, *n* = 7), respectively

Not only cytoplasmic Ca^2+^ and Mg^2+^ but also pH plays a role in activation of the channels, which was confirmed by the experiments with different cytoplasmic pH 7, 6.5, and 6.0 (Fig. 7). The decrease in the cytoplasmic pH from 7 to 6.5 caused reduction in the open probability from 0.26 to 0.16 (Fig. 7d, upper histogram and middle histogram, respectively), but did not affect the current amplitude, which at pH 7 and pH 6.5 was similar (this value obtained from Gaussian fittings of histograms reached 8.07 and 8.01 pA, respectively). After the decrease in the cytoplasmic pH to 6.0, almost complete reduction of the open probability of the channels was observed (0.005, Fig. 7d, lower histogram). In turn, an increase in the vacuolar pH from 5 (Fig. 3) to 7 or 8 (Fig. 8a, b) did not reduce the channel activity, but caused a decrease in single channel conductance, which at −80 mV reached: 95.5 ± 1.37 pS (*n* = 6) in pH 5, 80.1 ± 1.3 pS (*n* = 9) in pH 7 and 72.9 ± 1.8 pS (*n* = 7) in pH 8.

## Discussion

NO_3_^−^ transport through the tonoplast in plants is still poorly understood. The role of this transport is assigned to some members of CLC transporters. Among the wide family of these transporters, some are located in the tonoplast, like OsCLC1 and OsCLC2 from *Oryza sativa* (Diedhiou and Golldack 2006; Nakamura et al. 2006), GmCLC1 from *Glycine max* (Li et al. 2006), and AtCLCa-c and AtCLCg from *Arabidopsis thaliana* (De Angeli et al. 2006; Lv et al. 2009; von der Fecht-Bartenbach et al. 2010). The activity of the AtCLCa transporter recorded in *Arabidopsis thaliana* (De Angeli et al. 2006) proved that this protein acts as a NO_3_^−^/H^+^ exchanger involved in accumulation of nitrate in the vacuole. A characteristic feature of this transporter was NO_3_^−^ over Cl^−^ selectivity and capability of nitrate transport into the vacuole. Channels recorded in *Physcomitrella* possess similar properties—the ability to carry NO_3_^−^ currents from the cytoplasm to the vacuole (Figs. 1, 3), and NO_3_^−^ over Cl^−^ selectivity (Fig. 4). However, the nitrate channels in *Physcomitrella* differed from the NO_3_^−^/H^+^ exchanger AtCLCa from *Arabidopsis* with respect to dependence of the currents on pH. Changes in pH in *Arabidopsis* affect the reversal potential and magnitude of AtCLCa currents (De Angeli et al. 2006), whilst in *Physcomitrella*, the increase in the vacuolar pH caused a decrease in currents flowing through the channels, but not a shift in the reversal potential (Fig. 8). The lack of the influence of the pH gradient on the reversal potential was supported by recordings carried out at the symmetrical concentration of NO_3_^−^, where the reversal potential was close to zero at a pH gradient equal to 2 units (Figs. 1c, 3c, f). Trying to qualify the nitrate-permeable channels from *Physcomitrella,* we decided to compare them to AtCLCa. In silico searching carried out in the NCBI databases (http://www.ncbi.nlm.nih.gov/) allowed finding proteins from *Physcomitrella* similar to AtCLCa (UniProtKB accession number P92941), among which the highest identity (49 %) was exhibited by a protein with a GenBank accession number EDQ78881. The same protein shares 49–54 % similarity with AtCLCb, AtCLCc, and AtCLCg (UniProtKB accession numbers of these proteins are P92942, Q96282, and P60300, respectively). We also found high similarity of some proteins from *Physcomitrella* to vacuolar OsCLC1 and OsCLC2 channels from *Oryza sativa* (GenBank accession numbers of these proteins are BAB97267 and BAB97268, respectively). Again, a protein with a GenBank accession number EDQ78881 exhibited the highest similarity (57 %) to OsCLC1 and OsCLC2, but also two other proteins (EDQ52731, EDQ63773) with high similarity (49–51 %) were found with respect to the proteins from *Oryza sativa*. In silico searching was also extended to the nitrate transporter located in the tonoplast of *Arabidopsis*—ATNRT2.7 (UniProtKB accession number Q9LYK2). The results showed six nitrate transporters in *Physcomitrella* (GeneBank accession numbers: BAF42659, BAD00099, BAF42657, EDQ67083, BAF42658, EDQ67085) whose similarity to ATNRT2.7 amounts to 45–48 %.

The results of in silico research indicated that some proteins from *Physcomitrella* were similar to CLC-type proteins from *Arabidopsis* and *Oryza* and also to another kind of a nitrate transporter from *Arabidopsis*—ATNRT2.7, but it is still not possible to determine the activity of which protein was recorded in our patch-clamp experiments carried out on wild-type plants. Although the known genome of *Physcomitrella* gives a possibility to make heterologous expression of selected ion transporters, this was not the aim of our study, which was focused on studying of NO_3_^−^permeability of the native tonoplast.

Interestingly, the NO_3_^−^ permeable channels from *Physcomitrella* share some features with SV type channels also recorded in this plant (Koselski et al. 2013). Both channels are activated by a similar concentration of cytoplasmic Ca^2+^ of ca. 10 µM (as shown in our Fig. 5 and by Koselski et al. 2013 in Fig. 4). Another common feature of the channels is their dependence on Mg^2+^ and pH. As in the case of NO_3_^−^ permeable channels from *Physcomitrella*, SV channels from higher plants are activated by cytoplasmic Mg^2+^ (Allen and Sanders 1996; Pei et al. 1999; Carpaneto et al. 2001) and suppressed by low cytoplasmic pH (Schulz-Lessdorf and Hedrich 1995). The activity of SV channels in our experiments can be discussed, because the main cation which was used—BTP, has a similar diameter ~0.7 nm (Franciolini and Nonner 1994) as the pore of the SV channel at its narrowest place (Pottosin and Schönknecht 2007). The residual permeability of the examined channels to BTP could be the cause of the discrepancy between E_NO3_ and the reversal potential obtained in the NO_3_^−^ gradient (Fig. 2f). However, in such conditions, a substantial difference occurs between the measured reversal potential (−34.9 mV) and E_BTP_ (16.2 mV). Moreover, we showed that replacement of Na^+^ with BTP caused complete reduction of the currents flowing through SV channels (Figs. 1, 2), indicating that BTP does not pass through these channels. Taking into account the strong outward rectifying behaviour of SV channels, it is doubtful that such channels would allow BTP to pass from the vacuole to the cytoplasm, since at a lower concentration of this cation on the vacuolar side (101 mM in comparison to 160 mM added on the cytoplasmic side, Fig. 3a) we recorded mainly negative inward currents.

The discrepancy between the measured reversal potential and E_NO3_ can indicate contribution of other than NO_3_^−^ ions in the recorded currents. One of the ions that could affect the reversal potential was Cl^−^ added in a symmetrical concentration (8 mM) on both sides of the tonoplast (Fig. 3). The permeability of the channels from *Physcomitrella* to Cl^−^ was confirmed by recordings of the channel activity in the absence of NO_3_^−^ (Fig. 4a). In the same experiment, replacement of Cl^−^ to NO_3_^−^ on the cytoplasmic side of the tonoplast (Fig. 4b) proved that the channels were approximately three times more permeable to NO_3_^−^ than Cl^−^. In the experiments verifying the channel selectivity (Fig. 3), the low permeability of the channel to Cl^−^ and its much lower concentration than NO_3_^−^, allowed us to assume that the effect of Cl^−^ on the reversal potential, if it occurs, should not be higher than a few millivolts. A much higher difference between E_NO3_ and the reversal potential was observed in our experiments (16.7 mV).

Up to now, there have been insufficient data showing the activity of single NO_3_^−^ permeable channels in the plant vacuole and it is hard to classify the channels from *Physcomitrella*. Only some of the regulatory mechanisms of *Physcomitrella* channels are common with these described earlier in vacuolar anion channels from different plants. For instance, strong dependence on cytoplasmic Mg^2+^ and weaker dependence on some other divalent cytoplasmic cations (including Ca^2+^) were observed during patch-clamp recordings of vacuolar anion channels from the liverwort *Conocephalum conicum* (Trebacz et al. 2007). These channels were also selective to NO_3_^−^ and, in comparison to other tested anions, the presence of NO_3_^−^ in the cytoplasm evoked the highest density of currents carried by the channels. Cytoplasmic Ca^2+^ plays a crucial role also in activation of vacuolar chloride channels recorded in the whole-vacuole configuration in a higher plant, *Vicia faba* (Pei et al. 1996). In this plant, vacuolar anion channels are activated by CDPK (Calcium-Dependent Protein Kinase), acting together with Ca^2+^. Cytoplasmic Ca^2+^ together with Zn^2+^ also activates chloride channels in tobacco vacuoles (Ping et al. 1992). As in the case of the NO_3_^−^ permeable channels from *Physcomitrella*, chloride channels in tobacco were silent during the activity of channels permeable to K^+^. The calcium-dependent mechanism of the H^+^/NO_3_^−^ antiport operates in the tonoplast of cucumber root cells (Migocka et al. 2013), where phosphorylation of a tonoplast antiporter involving Ca-dependent staurosporine-sensitive protein kinase may be involved in stimulation of the H^+^/NO_3_^−^ antiport. Another feature of the NO_3_^−^ permeable channels from *Physcomitrella* was the dependence of the channel conductance on cytosolic nitrate (Fig. 3f). A process of vacuolar anion selective channel regulation by chloride was found in *Beta vulgaris* (Plant et al. 1994), but in the case of these channels increasing vacuolar but not cytoplasmic chloride induced an increase in the inward currents carried by nitrate, acetate, and phosphate.

In this work, we studied dependence of NO_3_^−^ permeable channels from *Physcomitrella* on the cytoplasmic concentration of Ca^2+^, Mg^2+^ and pH—factors whose fluctuations play a role in some intracellular processes. Among the three factors mentioned, only Ca^2+^ operates in non-physiological concentrations, that is why participation of this ion in accumulation of NO_3_^−^ in the vacuole is questionable. Higher than the physiological cytoplasmic calcium concentration is also required for activation of vacuolar calcium-dependent channels from other plants, e.g. SV channels recorded in *Arabidopsis thaliana* in the presence of cytoplasmic Ca^2+^ exceeding 10 µM (Schulze et al. 2011) or SV channels from guard cells of *Vicia faba* being inactive at a calcium concentration lower than 10 µM (Pei et al. 1999). Some clues about the possibility of SV channel activation at lower and more physiological concentrations of cytoplasmic Ca^2+^ can be found in papers proving the increase in the channel activity as a result of (1) a synergistic effect of cytoplasmic Ca^2+^ and Mg^2+^ (Pei et al. 1999), (2) a decrease in vacuolar Ca^2+^ (Pottosin et al. 1997; Koselski et al. 2013), (3) an increase of cytoplasmic or vacuolar pH (Schulz-Lessdorf and Hedrich 1995), (4) addition of reducing agents (Carpaneto et al. 1999; Scholz-Starke et al. 2004). Activated by high and non-physiological concentrations of cytoplasmic Ca^2+^, channels are present not only in plant vacuoles. In the vacuole of yeast *Saccharomyces cerevisiae* Bertl and Slayman (1990) recorded cation-selective channels activated by cytoplasmic Ca^2+^ concentrations ≥1 mM. Similar to plant SV channels, these channels were also activated by reducing agents. Dependence of the vacuolar channels from *Saccharomyces cerevisiae* coded by a TRP-like (transient receptor potential) gene on the high 1 mM concentration of cytoplasmic Ca^2+^ was confirmed by Palmer et al. (2001).

The results of our study revealed that even at elevated concentrations of cytoplasmic Ca^2+^ (1 µM) and the presence of an antioxidant—DTT (Fig. 5e), whose effectiveness in activation of calcium- and voltage-dependent channels was confirmed in plant vacuolar channels (Carpaneto et al. 1999; Paganetto et al. 2001; Scholz-Starke et al. 2004), the NO_3_^−^ permeable channels from *Physcomitrella* remain closed. Although these studies revealed involvement of redox status in activation of the channels by cytoplasmic calcium, it is probable that another, so far unknown factor(s) can be essential for the in vivo functioning of the channel. The two other factors tested, Mg^2+^ and pH, affected the channel activity at the physiological range of the concentrations. Mg^2+^ and pH can cooperate in regulation of these channels. In a complex with ATP, Mg^2+^ is responsible for driving proton pumps and creating a proton-motive force through the cell membrane (by H^+^-ATPase) and the tonoplast (by V-ATPase) (Maeshima and Nakanishi 2002). For example, Hanstein et al. (2011) proved that the activity of H^+^-ATPase in maize decreased in the presence of Mg^2+^-free ATP and was restored after increasing the Mg^2+^ concentration. The influence of the free Mg^2+^ concentration on the stability and activity of V-PP_i_ase, another tonoplast H^+^ pump, was confirmed in mung bean vacuoles (Maeshima 1991). Some importance in Mg^2+^ and H^+^ balancing can be also assigned to the Mg^2+^/H^+^ vacuolar exchanger characterised in *Arabidopsis thaliana* (Shaul et al. 1999).

NO_3_^−^ permeable channels recorded in *Physcomitrella* share some similarities with aluminum-activated malate transporters (ALMTs). Two members of the ALMT family were identified in the plant vacuole. The first, AtALMT9, was initially characterised in the vacuole of *Arabidopsis thaliana* (Kovermann et al. 2007; De Angeli et al. 2013b) and then in *Vitis vinifera* (De Angeli et al. 2013a). The second member of ALMT recorded in *Arabidopsis* was AtALMT6 (Meyer et al. 2011). A common feature of NO_3_^−^ permeable channels from *Physcomitrella* and malate transporters is strong rectification of the currents in the same direction. Moreover, AtALMT6 is activated in the presence of 100 µM cytoplasmic calcium—a similar concentration of calcium ions activates NO_3_^−^ permeable channels from *Physcomitrella*. The patch-clamp measurements of AtALMT9 activity proved also that these transporters are chloride-permeable channels activated by cytoplasmic malate (De Angeli et al. 2013b). In contrast, there are no published data demonstrating nitrate fluxes through ALMTs. There is also another feature that differentiates the channels recorded in *Physcomitrella* from ALMTs—the much lower conductance of AtALMT9 (32 pS recorded in symmetrical 100 mM Cl^−^) with respect to channels from *Physcomitrella* (95.5 pS recorded in symmetrical 200 mM NO_3_^−^). It should be mentioned that much lower conductance (3 pS recorded in the presence of 100 and 10 mM malate/BTP on the cytoplasmic and vacuolar side, respectively) possesses an inward-rectifying vacuolar malate channel of a plant showing crassulacean acid metabolism (CAM), *Kalanchoë daigremontiana* (Hafke et al. 2003).

In conclusion, this study describes a possible pathway for nitrate transport from the cytoplasm to the vacuole, based on the activity of NO_3_^−^ permeable channels. Although accumulation of nitrate in the vacuole via CLC-type proteins has been proved earlier (De Angeli et al. 2006), the regulation of this process is still poorly understood. In this work, we focused on studying of the regulatory mechanism of NO_3_^−^ permeable channels from *Physcomitrella* by some factors involved in cell signalling (e.g. the level of cytoplasmic Ca^2+^) or in secondary transport through the tonoplast (facilitated by the pH gradient). We believe that this work fills a gap in our knowledge about vacuolar transport of nitrate in plants, especially concerning the functioning of nitrate-permeable channels.

### *Author contribution*

Mateusz Koselski conceived and designed research, conducted experiments, analyzed data, and wrote the main part of the manuscript. Halina Dziubinska participated in writing the manuscript and its reviewing. Aleksandra Seta-Koselska participated in preparation of plant material and reviewed the manuscript. Kazimierz Trebacz reviewed the manuscript and received a grant NCN (National Science Centre) no. 2013/09/B/NZ1/01052. All authors read and approved the manuscript.

## Electronic supplementary material

Below is the link to the electronic supplementary material.

Supplementary material 1 (PDF 178 kb)

## Abbreviations

ALMT: Aluminium-activated malate transporter
BTP: Bis-tris propane
CAM: Crassulacean acid metabolism
CDPK: Calcium-dependent protein kinase
ClC: Chloride channel
DTT: Dithiothreitol
EGTA: Ethylene glycol tetraacetic acid
E_NO3_: Reversal potential for nitrate
NRT: Nitrate transporter
SV: Slowly activating vacuolar channel
TRP: Transient receptor potential
VK: Vacuolar K^+^ channel
