# Supplementary material for: A nitrate-permeable ion channel in the tonoplast of the moss Physcomitrella patens
Source: Planta. 2015 Feb 1;241(5):1207–19. doi: 10.1007/s00425-015-2250-3 (PMC4412613; doi:10.1007/s00425-015-2250-3)
Supplement: Supplementary file 1 — Supplementary material 1 (PDF 178 kb) [file 425_2015_2250_MOESM1_ESM.pdf]

Supplementary material for the following article published in Planta

Title: A nitrate-permeable ion channel in the tonoplast of the moss *Physcomitrella patens*

Authors:

Koselski Mateusz, Dziubinska Halina, Seta-Koselska Aleksandra[1], Trebacz Kazimierz

Department of Biophysics, Institute of Biology and Biochemistry, Maria Curie-Skłodowska University, Akademicka 19, 20-033 Lublin, Poland

[1]Department of Plant Physiology and Biotechnology, Institute of Biotechnology, The John Paul II Catholic University of Lublin, Konstantynow 1i, 20-708 Lublin, Poland

Corresponding author:

Koselski Mateusz

Department of Biophysics, Institute of Biology and Biochemistry, Maria Curie-Skłodowska University, Akademicka 19, 20-033 Lublin, Poland

tel.: (48) 81 537 59 55, fax.: (48) 81 537 59 01

e-mail: mateusz.koselski@poczta.umcs.lublin.pl

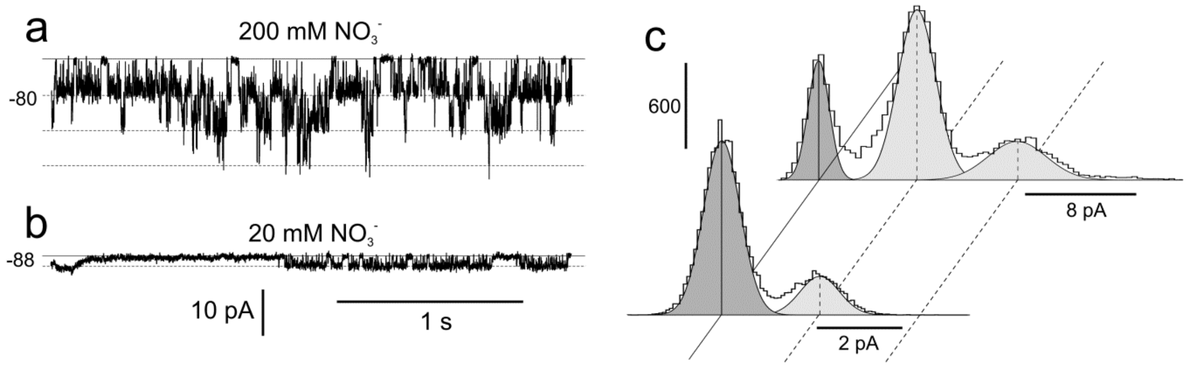

Dependence of the channel activity on the cytoplasmic HNO_3 concentration. **a** Cytoplasm-out recordings obtained at -80 mV in 200 mM HNO_3, 2 mM $CaCl_2$, 2 mM $MgCl_2$, pH 7 (buffered by 160 mM BTP) in the bath and 200 mM HNO_3^-, 2 mM $CaCl_2$, 2 mM $MgCl_2$, pH 5 (buffered by MES/TRIS) in the pipette. **b** Cytoplasm-out recordings obtained at -88 mV after replacement of the bath solution with 20 mM HNO_3, 2 mM $CaCl_2$, 2 mM $MgCl_2$, pH 7 (buffered by 16 mM BTP). **c** Amplitude histograms based on recordings from four patches obtained in conditions as in **a** (upper histogram), and **b** (lower histogram), respectively
